# Unilateral Corneal Insult Also Alters Sensory Nerve Activity in the Contralateral Eye

**DOI:** 10.3389/fmed.2021.767967

**Published:** 2021-11-15

**Authors:** Carolina Luna, Susana Quirce, Adolfo Aracil-Marco, Carlos Belmonte, Juana Gallar, M. Carmen Acosta

**Affiliations:** ^1^Instituto de Neurociencias, Universidad Miguel Hernández-CSIC, San Juan de Alicante, Spain; ^2^Instituto de Investigación Sanitaria y Biomédica de Alicante, San Juan de Alicante, Spain

**Keywords:** dry eye, sensory nerve activity, contralateral effects, corneal inflammation, corneal lesion

## Abstract

After the unilateral inflammation or nerve lesion of the ocular surface, the ipsilateral corneal sensory nerve activity is activated and sensitized, evoking ocular discomfort, irritation, and pain referred to the affected eye. Nonetheless, some patients with unilateral ocular inflammation, infection, or surgery also reported discomfort and pain in the contralateral eye. We explored the possibility that such altered sensations in the non-affected eye are due to the changes in their corneal sensory nerve activity in the contralateral, not directly affected eye. To test that hypothesis, we recorded the impulse activity of the corneal mechano- and polymodal nociceptor and cold thermoreceptor nerve terminals in both eyes of guinea pigs, subjected unilaterally to three different experimental conditions (UV-induced photokeratitis, microkeratome corneal surgery, and chronic tear deficiency caused by removal of the main lacrimal gland), and in eyes of naïve animals *ex vivo*. Overall, after unilateral eye damage, the corneal sensory nerve activity appeared to be also altered in the contralateral eye. Compared with the naïve guinea pigs, animals with unilateral UV-induced mild corneal inflammation, showed on both eyes an inhibition of the spontaneous and stimulus-evoked activity of cold thermoreceptors, and increased activity in nociceptors affecting both the ipsilateral and the contralateral eye. Unilateral microkeratome surgery affected the activity of nociceptors mostly, inducing sensitization in both eyes. The removal of the main lacrimal gland reduced tear volume and increased the cold thermoreceptor activity in both eyes. This is the first direct demonstration that unilateral corneal nerve lesion, especially ocular surface inflammation, functionally affects the activity of the different types of corneal sensory nerves in both the ipsilateral and contralateral eyes. The mechanisms underlying the contralateral affectation of sensory nerves remain to be determined, although available data support the involvement of neuroimmune interactions. The parallel alteration of nerve activity in contralateral eyes has two main implications: a) in the experimental design of both preclinical and clinical studies, where the contralateral eyes cannot be considered as a control; and, b) in the clinical practice, where clinicians must consider the convenience of treating both eyes of patients with unilateral ocular conditions to avoid pain and secondary undesirable effects in the fellow eye.

## Introduction

The ocular surface (OS) is innervated by different functional types of sensory neurons that not only evoke conscious sensations but also contribute to corneal tissue tropism and initiate protective motor and autonomic reflexes such as blinking and tearing (1–7). There are two types of nociceptor fibers innervating the cornea, namely, mechanonociceptors, which express Piezo2 channels and respond only to mechanical forces (8, 9), and polymodal nociceptors, which express a diversity of ion channels, such as TRPA1, TRPV1, ASIC, and Piezo2 that allow them to respond to a variety of stimuli applied on their receptive field, including mechanical forces, heating, and several irritant chemical substances (9–13). In the conjunctiva, low-threshold mechanoreceptors that evoke touch when stimulated have been also described (14, 15). The selective stimulation of these two populations of nociceptors in humans evokes sensations of irritation and pain, although with different qualities of the sensation (6). In humans, the selective activation of polymodal nociceptors has been described as the sensory arm to evoke the aforementioned protective tearing and blinking reflexes (1, 7). Additionally, cold thermoreceptors have been described in the cornea (10, 11, 16–19). Cold thermoreceptors express TRPM8 transducing channels and typically respond to decreases in temperature with different thresholds: canonical cold thermoreceptors with high background activity at normal corneal temperature and high sensitivity to cooling, and presumed cold nociceptors, that exhibit a low background activity and requires more intense cooling to increase its firing rate. Their selective stimulation in humans evokes sensations described as freshness/cold or dryness/pain, respectively, depending on the amplitude of temperature decrease (6). In addition, to evoke conscious sensations, the activity of cold thermoreceptors expressing TRPM8 channels contribute to the control of basal tearing and spontaneous blinking (3–5, 20).

We have previously shown that after unilateral OS inflammation or lesion, corneal sensory nerve activity and sensations are altered in the ipsilateral eye (21–26). As in other tissues, corneal nociceptors (specially polymodal nociceptors) are sensitized after lesion or during ocular surface inflammation (22–27). Nociceptor sensitization is characterized by an increase in spontaneous activity, reduction of the response threshold, and/or increased response to stimulation with stimuli of the same intensity (28). Additionally, corneal nociceptors also contribute to the inflammatory processes of the OS (a process known as neurogenic inflammation) (29, 30) by their release of pro-inflammatory neuropeptides as substance P and Calcitonin Gene-Related Peptide (CGRP) (31–34). The activity of cold thermoreceptors is decreased under inflammation (23, 24) through the inhibition of the TRPM8 channels by inflammatory mediators (35), and increased in chronic tear deficiency through changes in the Na+ and K+ channel expression and/or activity (25).

However, no previous studies have defined the changes in the sensory nerve activity at the contralateral eye nerves after unilateral nerve lesion or inflammation and if the putative change in their neural activity affects sensations only or also affects ocular tropism and protective reflexes. This is of special relevance because previous studies suggest bilateral changes in corneal sensitivity in patients with unilateral infectious keratitis (36), as well as bilateral changes in corneal nerve morphology and density in humans (37–42) and animals (43, 44).

In the present study, we recorded the impulse activity of mechano- and polymodal nociceptor and cold thermoreceptor nerves in guinea pigs, innervating the cornea of both eyes, under different experimental inflammatory or injury conditions (UV-induced photokeratitis, microkeratome surgery, and chronic tear deficiency) affecting only one eye, to answer the question of whether unilateral eye surface damage also modifies the nerve activity and sensitivity of the contralateral fellow eye. This information is important for the design of research protocols in the future, in which the use of contralateral eyes as controls should be excluded, and also to determine the convenience of applying bilateral treatment when unilateral ocular inflammation, infection, or injury affect one of the eyes.

## Materials and Methods

### Animals

Guinea pigs of both sexes, weighing 200–400 g at the beginning of the experiment, were used. The study was performed in accordance with the Association for Research in Vision and Ophthalmology (ARVO) Statement for the Use of Animals in Ophthalmic and Vision Research, the National Institutes of Health (NIH) Guide for the Care and Use of Laboratory Animals, the European Union Directive (2010/63/EU), and the Spanish regulations on the protection of animals used for research, following protocols approved by the Ethics Committee of the Universidad Miguel Hernández de Elche. The animals were kept in individual cages under a controlled day–night cycle with free access to food and water.

### Experimental Groups

The animals were distributed into four groups: (a) Control: A group of 42 animals without any experimental manipulation; (b) Unilateral UV-irradiation: A group of 9 animals subjected to unilateral UV irradiation of the OS; (c) Unilateral microkeratome corneal lesion: A group of 6 animals in which unilateral corneal nerve lesion was caused mechanically; (d) Unilateral main lacrimal gland excision: A group of 6 animals subjected to unilateral excision of the main lacrimal gland.

After the different experimental interventions, the animals were allowed to recover postoperatively and then housed individually under standard conditions in a certified animal facility. They were inspected daily for ocular inflammation, corneal epithelial defects or infections, as well as for abnormal behavior, and were treated accordingly. Before euthanizing the animal for the *ex vivo* electrophysiological recording of the electrical activity of corneal sensory nerves (see below), the ocular surface of both eyes was evaluated with a pocket slit lamp and the tear volume was measured.

#### Unilateral UV Irradiation

Under deep anesthesia (80 mg/kg ketamine and 4 mg/kg xylazine, i.p.), 254 nm UV-C radiation (1,000 mJ/cm^2^) was delivered for 49 min to one eye of the animal with a UV lamp (VL-4.C 230V 50/60 Hz; Vilber Lourmat, Marne-la-Vallée, France) placed at a distance of 17 cm from the eye. The animals were euthanized 48 h after the UV irradiation and both eyes were excised for electrophysiological recording. In a previous work, we showed that after the exposure to this intensity of UV radiation, mild clinical signs of inflammation and significant changes of the spontaneous and stimulus-evoked activity of the corneal sensory receptors were developed in the ipsilateral eye, maximal of 48 h after UV (24).

#### Unilateral Microkeratome Corneal Lesion

In the anesthetized guinea pigs (ketamine 50 mg/kg and xylazine 5 mg/kg, i.p.; topical 0.1% tetracaine, and 0.4% oxybuprocaine), a corneal flap of 4 mm diameter was cut at the mid-stromal depth in one eye using a custom-made microkeratome (Deriva Global, SL, Valencia, Spain) designed for the guinea pig eye (26). As the more prominent effects on the nerve activity in the lesioned eye were seen 24–48 h after the corneal lesion, in the present work, the animals were euthanized 24–48 h after the corneal lesion to study the corneal nerve activity in both eyes.

#### Unilateral Main Lacrimal Gland Excision

The animals were anesthetized with ketamine (90 mg/kg i.p.) and xylazine (5 mg/kg i.p.) for the unilateral removal of the main lacrimal gland. After performing an 8 mm skin incision on the temporal side, posterior to the lateral canthus, the fibrous capsule of the exorbital lacrimal gland was exposed and dissected, and the lacrimal gland was carefully excised (25). A drop of antibiotic (3 mg/ml tobramycin) was applied onto the surgical area before suturing the skin incision using a 6.0 braided silk suture. Before euthanasia and *ex vivo* recording, animals were housed for 4–11 weeks after surgery to fully develop corneal nerve alterations consecutive to chronic tear deficiency (25).

### Electrophysiological Recording and Analysis

The animals were killed with an i.p. overdose of sodium pentobarbitone, and their eyes, together with the bulbar and tarsal conjunctiva, were enucleated. The whole eye or the excised cornea (see below) was placed in a custom recording chamber superfused with the following physiological solution (in mM): 133.4 NaCl, 4.7 KCl, 2.0 CaCl_2_, 1.2 MgCl_2_, 16.3 NaHCO_3_, 1.3 NaH_2_PO_4_, and 7.8 glucose, gassed with carbogen to a pH = 7.4. The temperature of the perfusion solution was maintained at a basal temperature of 34°C with a homemade feedback-controlled Peltier device. Two types of preparations were used for the *ex vivo* recording of the corneal nerve activity, namely, the “whole eye” and the “isolated cornea” preparations (23, 24, 45). The “whole eye” preparation was particularly suitable for recording the polymodal and mechanosensory nociceptive units in the ciliary nerves, whereas, in the “isolated cornea” preparation, the activity of cold-sensitive units could be more easily identified [see (23) for a detailed schema of the preparations].

#### Recording Preparations

##### Whole Eye Preparation

The connective tissue and extraocular muscles in the back of the eye were carefully removed to expose and isolate the ciliary nerves traveling around the optic nerve. The eye was then placed in a recording chamber divided into two compartments by an elastomer-coated plastic wall (Sylgard 184; Dow Corning, Midland, Michigan, United States). The front of the eye including the cornea and the conjunctiva was introduced into a round perforation made in the center of the dividing wall to which the bulbar conjunctiva was pinned, thereby isolating the anterior segment from the back of the eye and the ciliary nerves, and preventing the direct exposure of the ciliary nerves to the solutions applied onto the corneal surface. The anterior compartment was continuously bathed with saline solution at 34°C and the back compartment was filled with warm mineral oil. The thin nerve filaments were teased apart from the ciliary nerve trunks and placed on an Ag-AgCl electrode for a monopolar recording of the unitary impulse activity of the axons innervating the cornea. Electrical signals from the recording electrode were fed to a 50-Hz noise eliminator (HumBug, Digitimer; Welwyn, United Kingdom) and then amplified and filtered (DAM50 amplifier; WPI, Sarasota, Florida, United States). Then, the signals passed through an analog-digital converter (CED Micro-1401; Cambridge Electronic Design, Cambridge, United Kingdom) and were stored in a personal computer (PC) with Spike2 software (v8.0; Cambridge Electronic Design) for the offline analysis.

##### Isolated Cornea Preparation

The corneas were excised around the limbus and then pinned to the bottom of a recording chamber continuously superfused with a physiological solution maintained at 34°C with a homemade Peltier device. To record the nerve terminal impulse (NTI) activity, a 50-μm-diameter glass micropipette filled with the physiological saline solution was applied gently to the corneal surface using a micromanipulator and then attached to the cornea by slight suction with a syringe. The electrical signals with respect to an Ag/AgCl pellet placed in the chamber were passed through a 50 Hz noise eliminator, amplified (AC preamplifier NL 103; Digitimer, Welwyn, United Kingdom), filtered (high pass 150 Hz, low pass 5 kHz; filter module NL 125; Digitimer), and then transferred to a PC with a Cambridge Electronic Design (CED) micro-1401 acquisition system and dedicated software, to be stored until the offline analysis.

#### Experimental Protocols

To study the electrophysiological activity of the corneal polymodal nociceptors, mechanonociceptors, and cold thermoreceptors, the following experimental protocols were performed:

##### Polymodal Nociceptors and Mechanonociceptors

After recording the electrical activity for 1 min to determine the spontaneous activity of the unit, the receptive field (RF) of the nociceptor fiber was located and mapped by mechanical stimulation with a fine paintbrush and a suprathreshold von Frey hair. Then, the mechanical threshold was determined with calibrated von Frey hairs of increasing force (range, 0.078–4 mN; Bioseb, Vitrolles, France). To ascertain its polymodality, the chemical sensitivity was tested with a low-flow jet of 98.5% carbon dioxide (CO_2_) and 1.5% air applied onto the RF for 30 s (CO_2_ pulse). The response to the temperature changes was eventually tested by changing the temperature of the perfusion solution for 30 s from 34°C up to 45°C (heating ramp) or down to 20°C (cooling ramp). At least 3 min were allowed between the different stimuli.

##### Cold Thermoreceptors

Nerve impulses originating at the single cold-sensitive nerve terminals were identified by their usually regular ongoing discharge at the basal temperature, which increased with cooling and decreased with warming. After recording the spontaneous activity at the basal temperature for at least 1 min, a cooling ramp from 34 to 20 °C was performed, followed by rewarming to 34 °C for 3 min. Then, a heating ramp to 45 °C was applied for 30 s before returning to the basal temperature.

#### Analysis of Sensory Nerve Activity

The characteristics of the spontaneous and stimulus-evoked impulse activity of the sensory nerves recorded in the control eyes, inflamed/lesioned/tear deficient eyes, and in contralateral eyes were analyzed offline using the Spike2 software. In the present work, the following parameters were calculated to compare the differences between the control, insulted, and contralateral eyes:

##### Polymodal Nociceptors

(a) The spontaneous activity was measured for 1 min at the beginning of the recording, before any intended stimulation (in impulses/s). (b) The mechanical threshold (in mN). (c) The latency of the impulse discharge evoked by the CO_2_ pulse, measured as the time (in s) elapsed between the onset of the gas pulse and the beginning of the impulse response. (d) The mean discharge rate of the response evoked by the CO_2_ pulse (in impulses/s). (e) Postdischarge, the mean discharge rate (in impulses/s) for 30 s after the end of the CO_2_ pulse.

##### Mechanonociceptors

(a) Spontaneous activity at the beginning of the recording, before any intended stimulation (in impulses/s). (b) Mechanical threshold (in mN).

##### Cold Thermoreceptors

(a) Ongoing activity measured for 1 min at a basal temperature of 34°C at the beginning of the recording (in impulses/s). (b) Cooling threshold, calculated as the decrease in temperature (in °C) during the cooling ramp from 34 to 20°C required to increase the mean frequency of discharge by 25% for 20 s before the ramp. (c) Peak frequency, the maximal value of the firing frequency (in impulses/s) reached during the cooling ramp. (d) Temperature change needed for the peak frequency, as the temperature change (in °C) is required to reach the peak frequency value during the cooling ramp.

#### Tear Volume Measurement

Tearing was measured in both eyes under stable environmental conditions (23°C temperature; 55% humidity) using commercial phenol red threads (Zone-Quick; Menicon, Tokyo, Japan) without topical anesthesia (23–25), before and after inducing the corneal insults (48 h after UV radiation, 24–48 h after the microkeratome lesion and 4 weeks after removal of the main lacrimal gland). The lower lid was gently pulled down, the folded 2 mm end of the thread was gently placed on the nasal palpebral conjunctiva, and the lid was then released. After a period of 30 s, the lower lid was, again, pulled down and the thread was gently removed. The entire length of the red-stained portion of the thread (in mm) was measured with a ruler under a stereomicroscope with an accuracy of 0.5 mm. The length of the red thread reflects both the tear volume in the conjunctival sac and the tear secretion over the 30 s of measurement.

#### Statistical Analysis of Data

The data were collected and processed for statistical analysis using the SigmaPlot software (SigmaPlot 11.0; Systat Software Inc, Point Richmond, California, United States). Unless otherwise stated, the data are expressed as mean ± SEM, with n being the number of explored units or eyes, as appropriate. The differences between the data from the different experimental groups were explored using a *t*-test or Mann-Whitney as needed. The differences between more than two groups were tested using one-way ANOVA or ANOVA on ranks, as needed. A *P*-value of 0.05 or less was considered significant.

## Results

### Unilateral UV Irradiation-Induced Mild Ipsilateral Inflammation, and the Sensitization of Nociceptors and Cold Thermoreceptor Inhibition in Both the Ipsilateral and Contralateral Eyes

#### Effects on the Ipsilateral, UV-Irradiated Eye

Forty-eight hours after the unilateral ocular exposure to 1,000 mJ/cm^2^ UV radiation, mild inflammation of the ocular surface (especially mild conjunctival hyperemia) could be observed only in the ipsilateral eye. The corneal nociceptors recorded in the ipsilateral eyes were sensitized, as reflected by the development of spontaneous activity (present in 3.1% of the 259 mechanonociceptor units from the control eyes and 18.5% of the 27 mechanonociceptor units recorded in the UV-irradiated eyes; *p* < 0.05, *Z*-test) and the significant decrease of the mechanical threshold of mechanonociceptors (0.64 ± 0.04 mN vs. 0.32 ± 0.03 mN, control vs. UV-irradiated eyes; *p* < 0.01, Mann-Whitney test). The spontaneous activity of polymodal nociceptors was significantly increased (6.6% in the control vs. 22% in the UV-irradiated eyes; *n* = 152 and 41, respectively; *p* < 0.01, Z-test), and also the discharge rate evoked by chemical stimulation was significantly higher (1.9 ± 0.2 imp/s vs. 3.4 ± 0.5 imp/s, control vs. UV-irradiated; *n* = 110 and 45 units, respectively, *p* < 0.01, Mann-Whitney test), suggesting the development of sensitization also in the polymodal nociceptor units recorded in the UV irradiated corneas. The spontaneous and cold-evoked activity of cold thermoreceptors was reduced in the UV-irradiated eyes (30.6 ± 1.3 imp/s vs. 18.9 ± 1.8 imp/s, control vs. UV-irradiated; *n* = 67 and 19, respectively; *p* < 0.001, *t*-test), while the cooling threshold and temperature to reach the peak frequency were not modified (data not shown).

#### Effects on the Contralateral, Non-irradiated Eye

In the contralateral eyes, no clinical signs of inflammation were found 48 h after UV irradiation. On the contrary, the activity of the different types of corneal nerves showed changes similar to those observed in the ipsilateral eye, although overall to a lesser degree. The mechanical threshold of mechanonociceptors was slightly reduced, although the difference was not statistically significant (Table 1). Similarly, the spontaneous activity of the mechanonociceptors and polymodal nociceptors did not change (Table 1). Twenty-five percent of the polymodal nociceptors exhibited spontaneous activity, which means the frequency was significantly higher than in the control eyes (Table 1), although no changes were observed in their response to CO_2_ pulses (Table 1).

**Table 1:** The spontaneous activity and stimulus-evoked responses of corneal nociceptors recorded in the control eyes and the eyes contralateral to UV irradiation, microkeratome lesion, or lacrimal gland removal (tear deficiency).

	**Control eyes**	**Contralateral eyes**		
		**Uv radiation**	**Microkeratome lesion**	**Tear deficiency**
**Mechanonociceptors**				
Mechanical threshold (mN)	0.64 ± 0.04	0.41 ± 0.12	0.42 ± 0.06^*^	0.58 ± 0.18
Spontaneous activity
Present in (%)	3,1%	0%	0%	0%
Spontaneous activity (imp/s)	0.7 ± 0.4	0.0 ± 0.0	0.0 ± 0.0	0.0 ± 0.0
*n*	275	11	42	8
**Polymodal nociceptors**				
Mechanical threshold (mN)	0.35 ± 0.03	0.35 ± 0.04	0.32 ± 0.03	0.27 ± 0.14
Spontaneous activity
Present in (%)	6.6%	25%^*^	12.5%	0%
Spontaneous activity (imp/s)	0.6 ± 0.3	1.1 ± 0.9	0.8 ± 0.3	0 ± 0
Response to CO_2_ pulse
Latency (s)	13.01 ± 0.77	10.68 ± 1.78	9.42 ± 1.85	16.80 ± 2.65
Mean discharge rate (imp/s)	1.94 ± 0.16	2.59 ± 0.47	2.12 ± 0.65	1.28 ± 0.51
Postdischarge (imp/s)	2.13 ± 0.50	2.86 ± 1.49	0.46 ± 0.15^*^	0.76 ± 0.39
*n*	167	19	17	6

* *p < 0.05, t-test or Mann-Whitney or Z-test (%), differences with control eyes*.

The ongoing activity at the basal temperature and peak response to the cooling ramps of cold thermoreceptors were not significantly modified in the contralateral eyes (Table 2), although the cooling threshold and the temperature change to reach the peak frequency were significantly reduced in comparison with the control eyes (Table 2).

**Table 2:** The spontaneous and stimulus-evoked activity of the corneal cold thermoreceptors recorded in the control eyes and the eyes contralateral to UV irradiation, microkeratome lesion, or lacrimal gland removal (tear deficiency).

	**Control eyes**	**Contralateral eyes**		
		**Uv radiation**	**Microkeratome lesion**	**Tear deficiency**
**Cold thermoreceptors**				
**Ongoing activity at 34** **°** **C**				
Present in (%)	100%	100%	100%	100%
Ongoing activity (imp/s)	9.0 ± 0.5	8.6 ± 2.2	6.8 ± 0.8^*^	12.5 ± 2.8^*^
**Response to cooling ramp from 34 to 20** **°C**				
Threshold (Δ°C)	−2.7 ± 0.2	−1.6 ± 0.1^*^	−2.3 ± 0.2	−1.9 ± 0.3
Peak frequency (imp/s)	30.6 ± 1.3	24.7 ± 4.1	29.6 ± 2.2	35.4 ± 5.5
Temp. change to peak frequency (Δ°C)	−6.1 ± 0.4	−4.0 ± 0.5^*^	−7.7 ± 0.9	−5.1 ± 0.6
*n*	67	14	24	10

* *p < 0.05 t-test, differences with control eyes*.

#### Differences Between Nerve Activity and Tear Volume of UV-Irradiated and Contralateral Eyes

**Figure 1:**
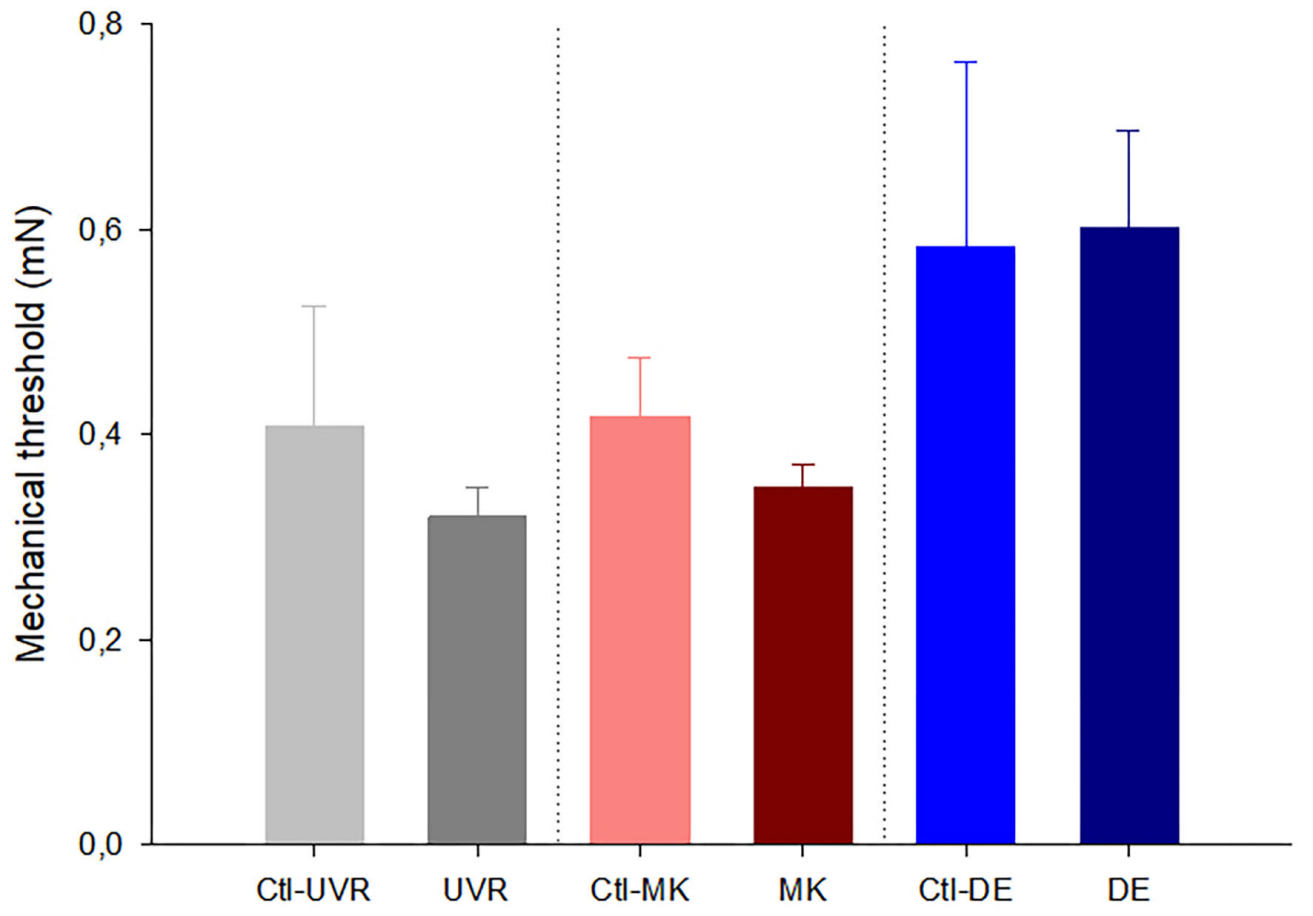
Effects of unilateral UV irradiation, microkeratome lesion, and main lacrimal gland removal on the mechanical threshold of the mechanonociceptor units recorded in the ipsilateral and contralateral (denoted by “Ctl” label) eyes. The data are presented as mean ± SEM. No significant differences were found between the data from the ipsilateral and contralateral eyes inside each group, Mann-Whitney test.

**Figure 2:**
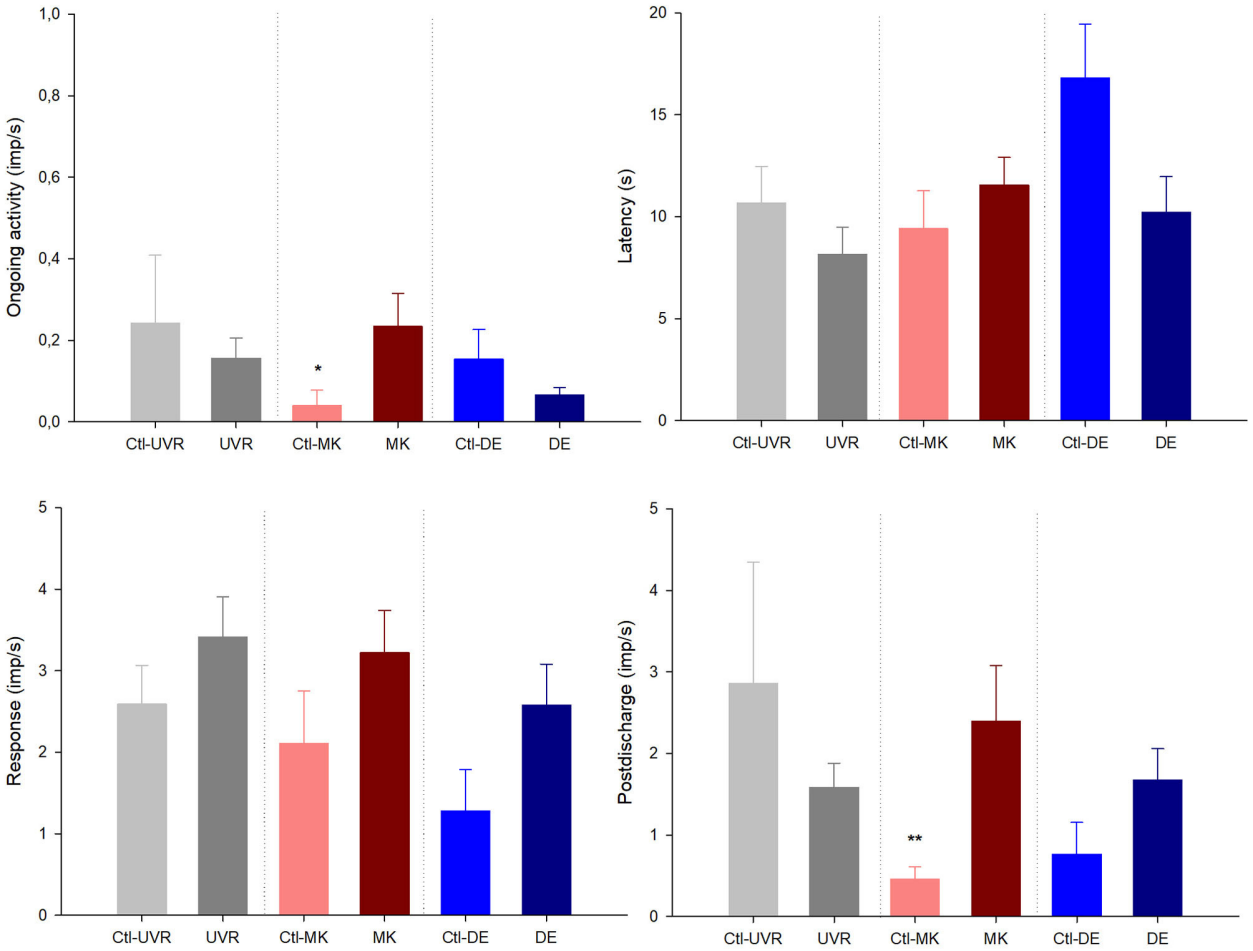
The effects of unilateral UV irradiation, microkeratome lesion, and main lacrimal gland removal on the ongoing activity and the response to the chemical stimulation with carbon dioxide (CO_2_) of the polymodal nociceptor units recorded in the ipsilateral and contralateral (denoted by “Ctl” label) eyes. The data are presented as mean ± SEM. *p < 0.05, **p < 0.01, *t*-test or Mann-Whitney, as needed, the differences between the data from the ipsilateral and contralateral eyes.

**Figure 3:**
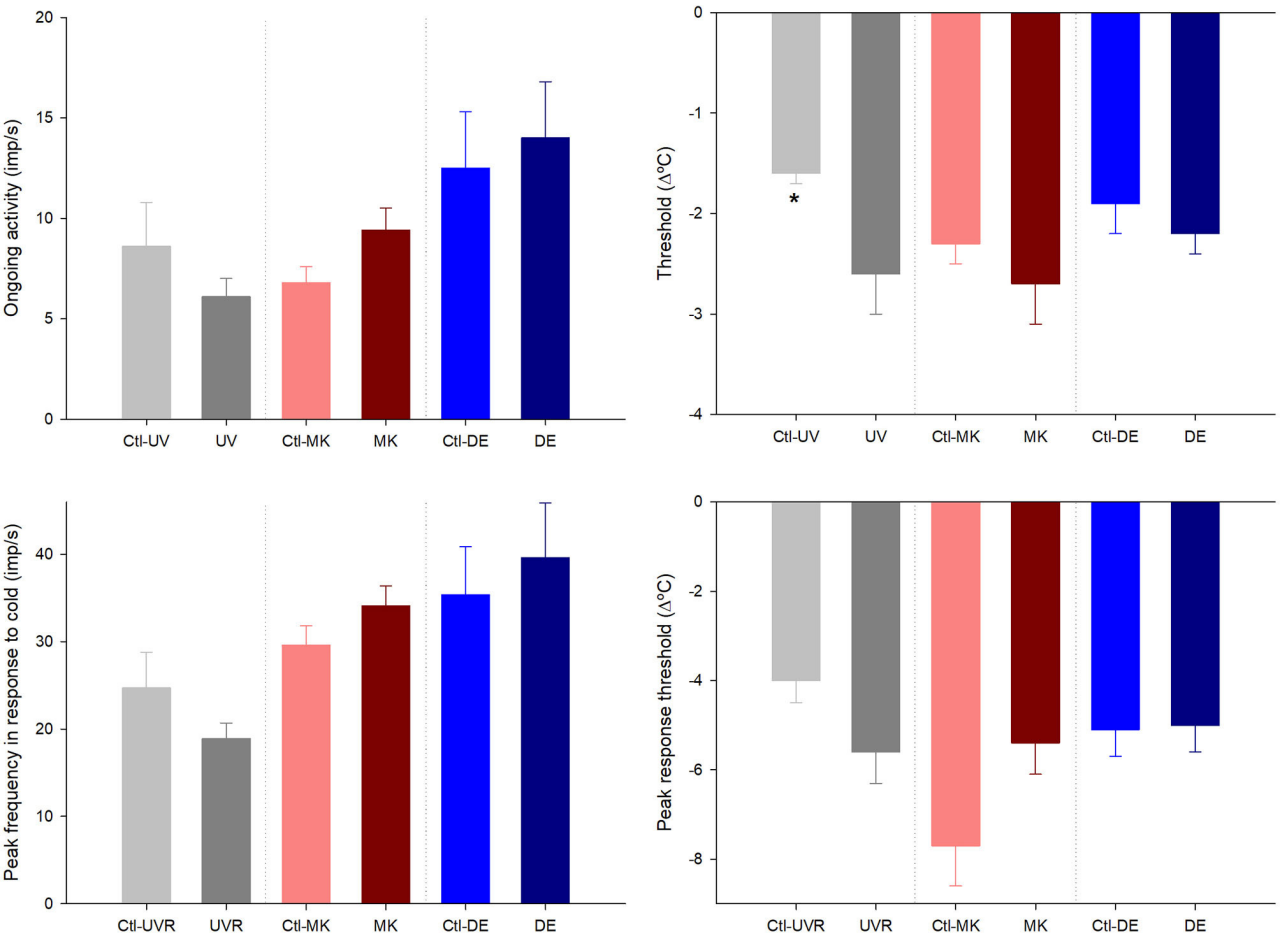
Effects of unilateral UV radiation, microkeratome lesion, and main lacrimal gland removal on the ongoing activity and the response to cooling ramps of the cold thermoreceptor units recorded in the ipsilateral and contralateral (denoted by “Ctl” label) eyes. The data are presented as mean ± SEM. *p < 0.05, *t*-test or Mann-Whitney, the differences between the ipsilateral and contralateral data inside each group.

When comparing the activity of the mechanonociceptors (Figure 1), polymodal nociceptors (Figure 2), and cold thermoreceptors (Figure 3) from the UV-irradiated and contralateral eyes, no significant differences were found. The tear volume was not significantly affected in neither the irradiated nor in the contralateral eyes when compared with the control (10.6 ± 0.7m, *n* = 42; 12.7 ± 1.9 mm, *n* = 9; 11.3 ± 1.3 mm, *n* = 9; control, UV-irradiated eyes and contralateral, respectively; *p* > 0.05, one-way ANOVA).

### Unilateral Corneal Microkeratome Lesion-Induced Ipsilateral Inflammation and the Sensitization of Nociceptors in Both the Ipsilateral and Contralateral Eyes

#### Effects on the Ipsilateral, Microkeratome Lesioned Eye

Twenty-four to 48 h after the unilateral corneal lesion with the microkeratome, a mild inflammation of the ocular surface (especially mild conjunctival hyperemia) was developed in the ipsilateral eye. The corneal surgical lesion with a microkeratome induced the sensitization of corneal nociceptors. The mechanical threshold of mechanonociceptors was significantly reduced (0.64 ± 0.04 vs. 0.35 ± 0.0.2 mN, control vs. lesioned eyes; *n* = 275 and 48, respectively; *p* < 0.05, Mann-Whitney test), and the response to the CO_2_ pulses of polymodal nociceptors was significantly increased (1.9 ± 0.2 vs. 3.4 ± 0.5 imp/s, control vs. lesioned; *n* = 110 and 33, respectively; *p* < 0.01, Mann-Whitney test). The spontaneous and stimulus-evoked activity of the cold thermoreceptors of the contralateral eyes presented values similar to that of the control eyes (data not shown).

#### Effects on the Contralateral, Non-lesioned Eye

The mechano-nociceptors of the contralateral eyes presented a significantly lower mechanical threshold, although they did not develop spontaneous activity (Table 1). The spontaneous and stimulus-evoked activity of the polymodal nociceptors was not significantly affected or slightly reduced in the contralateral eyes (Table 1). Similarly, the spontaneous and cold-evoked activity of the cold thermoreceptor of the eyes contralateral to the microkeratome lesion was similar to the control or slightly reduced (Table 2).

#### Differences Between Nerve Activity and Tear Volume of Microkeratome Lesioned and Contralateral Eyes

No differences were noticed in the mechanical threshold of the mechano-nociceptors recorded in the lesioned and contralateral (Figure 1). Regarding polymodal nociceptors, the spontaneous activity and postdischarge to CO_2_ stimulation were statistically lower in the contralateral than the lesioned eyes (Figure 2). No differences were found when comparing the spontaneous and stimulus-evoked activity of cold thermoreceptors (Figure 3).

No changes in tear volume were found in the lesioned and contralateral eyes compared with the control (10.6 ± 0.7 mm, *n* = 42; 7.5 ± 0.9 mm, *n* = 6; 10.8 ± 2 mm, *n* = 6; control, lesioned and contralateral eyes, respectively; *p* > 0.05, one-way ANOVA).

### Unilateral Lacrimal Gland Removal Decreased Tear Volume and Sensitized Corneal Cold Thermoreceptors in Both the Ipsilateral and Contralateral Eyes

#### Effects on the Ipsilateral Eye

Four weeks of tear deficiency induced by the unilateral removal of the main lacrimal gland induced signs of mild OS inflammation only in the operated side, with mild conjunctival hyperemia in all the operated guinea pigs and occasional mild corneal punctate after fluorescein staining. The tear volume was significantly reduced in the operated side and also in the contralateral eye, although to a lesser extent (see below Differences between the nerve activity and tear volume of the ipsilateral and contralateral eyes for details). The mechanical threshold of the mechanonociceptors was not modified 4 weeks after the lacrimal gland ablation (0.64 ± 0.04 vs.0.6 ± 0.09 mN, control vs. tear-deficient eyes; *n* = 167 and 27, respectively; *p* > 0.05, Mann-Whitney test). The spontaneous activity did not change (0.6 ± 0.3 imp/s vs.0.6 ± 0.4 imp/s, control vs. tear-deficient eyes; *n* = 259 and 27, respectively) and the response to CO_2_ pulses (1.9 ± 0.2 vs. 2.6 ± 0.5 imp/s, control vs. lesioned; *n* = 110 and 14, respectively) of polymodal nociceptors was slightly increased, although the differences were not statistically significant. On the other hand, the spontaneous activity at the basal temperature (9 ± 0.5 vs. 14 ± 2.8 imp/s, control vs. tear-deficient eyes; *n* = 67 and 8, respectively; *p* < 0.01, *t*-test) and stimulus-evoked activity (peak frequency to cooling ramps: 30.6 ± 1.2 vs. 39.6 ± 6.3 imp/s, control vs. tear-deficient; *p* < 0.05, *t*-test) of the cold thermoreceptors of the operated side was significantly higher than in the control eyes, with no changes in the cold response thresholds (data not shown).

#### Effects on the Contralateral Eye

Similar to the ipsilateral eyes, the activity of the mechanonociceptors and polymodal nociceptors was not significantly modified in the eyes contralateral to lacrimal gland removal (Table 1), except for the small but significant higher spontaneous activity of the polymodal nociceptors compared with those of the naïve, control eyes (Table 1). Similarly, a higher value of spontaneous activity was the only significant difference found in the activity of the cold thermoreceptors recorded in the contralateral eyes (Table 2).

#### Differences Between the Nerve Activity and Tear Volume of the Ipsilateral and Contralateral Eyes

No significant differences were found when comparing the spontaneous and stimulus-evoked activity of the mechanonociceptors (Figure 1), polymodal nociceptors (Figure 2), and cold thermoreceptors (Figure 3) recorded in the eyes contralateral and ipsilateral to lacrimal gland removal.

As expected, according to previous work (25), 11 weeks after the unilateral lacrimal gland excision, tear volume was significantly decreased in both eyes (10.6 ± 0.7, 1.3 ± 0.4, 4.0 ± 1.0 mm; control, ipsilateral and contralateral eyes, respectively, *n* = 6; *p* < 0.001, ANOVA on ranks, with Dunn's *post hoc* test). The tear volume reduction was significantly larger on the ipsilateral side than on the contralateral side (*p* < 0.05, Mann-Whitney test).

## Discussion

For the first time, the present results show the development of the same changes in the sensory nerve activity in both eyes after the experimental unilateral inflammation or lesion of the OS in only one eye. Corneal nerve activity is altered in the corneas of the contralateral side, although the magnitude of those changes was usually smaller than in the ipsilateral side and does not always achieve a statistically significant level. Mild corneal inflammation produces a sensitization of nociceptors and an inhibition of the activity of the cold thermoreceptors (23, 24), and the surgical lesion of corneal nerves produces a sensitization of nociceptors (26) and only small changes in the activity of cold thermoreceptors. Chronic tear deficiency is characterized by the inflammation of the OS and is known to produce nerve damage that can lead to corneal neuropathy (46). This condition also induces the sensitization of corneal sensory nerves, being the cold thermoreceptors the type of corneal fibers more affected by tear deficiency (25). The present results show that even when only one eye is primarily affected by inflammation or injury, the corneal sensory nerves of the contralateral side do not behave like those of the naïve eyes, as their spontaneous and/or stimulus-evoked activity is altered in the same way as in the lesioned side, although sometimes to a lesser degree.

In the present work, three different types of insults were performed to only one eye of the experimental animals. Among these three types of OS damage, the UV-induced corneal inflammation and chronic tear deficiency induced by lacrimal gland removal were the models that produced more significant changes in the activity of the corneal sensory receptors in the contralateral eye, while the corneal nerve damage produced by the unilateral corneal surgery presented fewer effects on the contralateral sensory nerve activity. The level of contralateral effects seems to depend on the degree of the inflammation induced in the ipsilateral eye, such that the greater the inflammation in the affected eye, the greater the contralateral effects.

The results support the idea that the contralateral effects are mostly due to an interaction between the nervous and the immune system as some authors have pointed out and as we will discuss here. One possible explanation for the contralateral effects would be the existence of innervation from the contralateral trigeminal ganglion, the trigeminal nuclei at the brainstem, or even at the superior levels of the central nervous system. It was described that there are a small number of ocular nerve fibers that travel to the contralateral trigeminal ganglion (47) and also that the central projections of some trigeminal ganglion neurons project to both the ipsilateral and contralateral trigeminal brainstem nuclei (48). These information crossing would explain, at least in part, how the altered sensory nerve activity arising at the damaged eye would affect the activity in the neurons processing the sensory information from the contralateral side.

Another possible explanation is that the contralateral effects are mediated by the existence of neuro-immune interactions. Some authors found evidence of a sympathetic inflammatory response in the contralateral side after unilateral inflammation or lesion, whose results support an interaction between the nervous and the immune system in the ocular tissues. In experimentally induced unilateral glaucoma, there is an activation of the macro- and micro-glia in both retinas, explained by an immune response after the breakdown of the blood-retinal barrier of the experimental eye (49–52).

Numerous previous works have studied the morphology and density of corneal nerves contralateral to a unilateral infection or injury. Almost all of them show that after unilateral damage, there is not only a decrease in the density of the subbasal nerves in the ipsilateral but also in the contralateral side. In patients with unilateral herpes simplex keratitis or herpes zoster ophthalmicus, there is a decrease in the corneal subbasal plexus nerve density in the affected eye and also in the contralateral eye (39, 40, 42), which explains the reduction of the contralateral corneal sensitivity (36). Moreover, there is also a bilateral increase in the dendritic cell density, which correlates with the decrease of the corneal subbasal nerves (37, 38, 40, 41), as well as in the levels of the pro-inflammatory cytokines in tears in patients with unilateral bacterial keratitis (43, 53). Several authors have also observed an increase of chemokines like MCP-1 in the aqueous humor in the contralateral eye after unilateral cataract surgery (54), although it has been suggested that the increase of MCP-1 is produced only in diabetic patients (55). In mice, after a unilateral surgical axotomy of the ciliary nerves or penetrating keratoplasty, there is also a decrease in the contralateral subbasal nerves (43, 56). After surgery, there is a bilateral release of substance P that has been proposed to abolish the immune privilege of both eyes (56) through disabled T regulatory cells (57). It has been also described that after corneal alkali burn, pro-inflammatory cytokines also increase Substance P and the expression of its receptor in the contralateral trigeminal ganglion, which supports the idea that substance P seems to be involved in the contralateral propagation of inflammation through corneal sensory nerves (30, 58).

Further supporting this, Guzman et al. (44) showed that unilateral corneal injury affects the contralateral ocular surface mucosa. After an injury, the sensory nerves of the OS are damaged, thus producing a neurogenic inflammatory reflex that is initiated in the injured eye through the activation of polymodal nociceptive sensory nerves expressing the TRPV1 channel. Subsequently, a neurogenic reflex is produced in both eyes, including the release of substance P that is the effector of the contralateral inflammatory response (44). Epithelial cells, DCs, and T cells express functional substance P receptors, and this neuropeptide exerts numerous proinflammatory functions (59) playing an important role in the ocular surface epithelial barrier function and DC pathophysiology (60).

In summary, the activation and sensitization of ipsilateral polymodal nociceptors after unilateral corneal nerve lesion or inflammation would produce the bilateral release of the substance P. The increase of substance P and proinflammatory substances in the contralateral ocular surface will produce an inflammation-like condition in the contralateral eye that would explain the changes of nerve activity that we have found in the present work, that is, the sensitization of corneal nociceptors and the reduced activity of cold thermoreceptors (23, 24). In the case of the unilateral ablation of the main lacrimal gland, which produces mainly an increase in the activity of cold thermoreceptors and has fewer effects on corneal nociceptors (25), it should be noticed that despite removing the gland of only one side, there is also a significant decrease in the tear volume in the contralateral eye, which could contribute to the effects observed in the activity of the contralateral cold thermoreceptors. However, we can only speculate if there is an immune-mediated effect on the contralateral nerves first and consequently, a decrease in tear volume or vice versa, that is, the chronic reduction of tearing in the contralateral eye led to changes in nerve activity. Although guinea pigs show delayed epithelial wound healing in both the ipsilateral and contralateral eyes after lacrimal gland removal in only one side (61), we only can speculate whether it is due to the chronic decrease in tear secretion, as no delay was observed in the microkeratome lesioned corneas (unpublished data), where there are no changes in tear secretion. Also, in the inflammatory and corneal lesion models used in the present work, there were no changes in the tear volume of either eye, although there were significant effects on the nerve activity in the contralateral eye, which are most probably mediated by neuro-immune interactions. Although Fakih et al. (62) did not study the effects on the contralateral side, in their mice models of tear deficiency, there is also an increase in pro-inflammatory markers and immune cells in the ipsilateral trigeminal ganglion and trigeminal brainstem nuclei 21 days after the surgery, indicating neuronal and microglial markers in the trigeminal brainstem and indicating how the effects of this pathology develop and maintains.

In conclusion, the ocular surface lesion and, especially, inflammation affect the activity of the unilateral corneal sensory receptors and also produces similar effects, although to a lesser degree, in the contralateral eye. The development of changes in the corneal nerve activity in the contralateral eye explains the development of ocular discomfort and pain sensation in the contralateral eye, which may not present any clinical sign. This has to be considered not only in experimental science, because the contralateral eye cannot be considered as a control, but also in the clinic. Even when only one eye has been affected by inflammation, infection, or injury, the pertinence of treating both eyes must be considered to avoid pain and other unwanted effects on the fellow eye.

## Data Availability Statement

The raw data supporting the conclusions of this article will be made available by the authors, without undue reservation.

## Ethics Statement

The animal study was reviewed and approved by Ethics Committee of the Universidad Miguel Hernández de Elche.

## Author Contributions

CL and SQ did the experiments and analyzed the data. CL, SQ, JG, and MA interpreted the data. JG and MA conceived, designed, and supervised the work. MA wrote the manuscript. All authors reviewed and edited the manuscript. All authors contributed to the article and approved the submitted version.

## Funding

This work was funded by the Spanish Agencia Estatal de Investigación and the European Regional Development Fund Grants SAF2017-83674-C2-1-R, SAF2017-83674-C2-2-R, and PID2020-115934RB-I00 funded by MICIN/AEI/1013039/5011100011033; the Generalitat Valenciana Excellence Program grant PROMETEO/2018/114 and Predoctoral Fellowship BES-2015-072638 from AEI (SQ). The APC was funded in part by Universidad Miguel Hernández de Elche.

## Conflict of Interest

The authors declare that the research was conducted in the absence of any commercial or financial relationships that could be construed as a potential conflict of interest.

## Publisher's Note

All claims expressed in this article are solely those of the authors and do not necessarily represent those of their affiliated organizations, or those of the publisher, the editors and the reviewers. Any product that may be evaluated in this article, or claim that may be made by its manufacturer, is not guaranteed or endorsed by the publisher.
